# The power of monitoring: optimizing survey designs to detect occupancy changes in a rare amphibian population

**DOI:** 10.1038/s41598-017-16534-8

**Published:** 2017-11-28

**Authors:** Izabela M. Barata, Richard A. Griffiths, Martin S. Ridout

**Affiliations:** 1 Durrell Institute of Conservation and Ecology, School of Anthropology and Conservation, University of Kent, Canterbury, Kent, CT2 7NR UK; 2 National Centre for Statistical Ecology, School of Mathematics, Statistics and Actuarial Science, University of Kent, Canterbury, Kent, CT2 7NF UK

## Abstract

Biodiversity conservation requires reliable species assessments and rigorously designed surveys. However, determining the survey effort required to reliably detect population change can be challenging for rare, cryptic and elusive species. We used a tropical bromeliad-dwelling frog as a model system to explore a cost-effective sampling design that optimizes the chances of detecting a population decline. Relatively few sampling visits were needed to estimate occupancy and detectability with good precision, and to detect a 30% change in occupancy with 80% power. Detectability was influenced by observer expertise, which therefore also had an effect on the sampling design – less experienced observers require more sampling visits to detect the species. Even when the sampling design provides precise parameter estimates, only moderate to large changes in occupancy will be detected with reliable power. Detecting a population change of 15% or less requires a large number of sites to be surveyed, which might be unachievable for range-restricted species occurring at relatively few sites. Unless there is high initial occupancy, rare and cryptic species will be particularly challenging when it comes to detecting small population changes. This may be a particular issue for long-term monitoring of amphibians which often display low detectability and wide natural fluctuations.

## Introduction

The global biodiversity crisis has driven the development of increasingly sophisticated databases, such as the Living Planet Index^1^ and the IUCN Red List for Threatened Species^2^, which require reliable baseline information on species, habitats and population trends. Although monitoring data are of increasing value to conservation managers, population and status assessments are currently limited by the lack of data^3^, resulting in poor evidence for conservation practitioners. Monitoring programmes must inform decision-making through the application of reliable survey design and statistical analysis – otherwise they will be an ineffective use of resources. Conservationists must therefore develop projects with clear objectives^4^ and provide appropriate sampling designs^5,6^ with sufficient statistical power to reliably describe population trends^7–9^. Nonetheless, issues of sampling design are widely ignored and remain a challenge for species monitoring and modelling^10^.

Occupancy modelling is increasingly being applied in monitoring programmes to assess the determinants of population changes for different taxonomic groups^11,12^. Occupancy models estimate site occupancy and detection probabilities in an unbiased way^13,14^ and occupancy may also be used as a proxy for abundance^6^. Although sampling designs for occupancy models have been explored theoretically^15–18^, few studies have used empirical data to investigate the survey effort required for the reliable inference of absence^19–21^ or to explore the precision and accuracy of occupancy estimates^22–24^. In the context of occupancy monitoring, studies have also considered statistical power using empirical data^8,24–29^. Statistical power considers the number of samples, variability in the data and the expected rate of change^30^ to evaluate the probability of detecting a change in the estimated parameter when that change actually occurs (e.g., increase or decrease in occupancy). Power analysis has long been recognized as a useful tool for study design, especially for the early stages of monitoring planning^4,7,18,31^.

Evaluating changes in populations at risk is particularly important in the case of amphibians, which are currently more threatened than birds or mammals and show accelerating rates of extinction^32^. However, amphibians are often rare, cryptic or elusive and can display considerable natural population fluctuations^33^, which can make long-term monitoring difficult. Significant advances in amphibian monitoring have been developed, such as the improvement of novel research methods (e.g., environmental DNA^34^), application of advanced data analysis (e.g., occupancy models^12^) and evaluation of national monitoring schemes (e.g., UK National Amphibian and Reptile Scheme^20^). Nonetheless, these developments are often limited by the availability of funding, which contributes further to difficulties in assessing population changes.

In this study we used patchily distributed bromeliads that are inhabited by a rare and threatened amphibian species, as a model system to assess sampling design and the statistical power associated with detecting population changes. The endemic frog *Crossodactylodes itambe* ^35,36^ is only found at the Itambe summit, southeastern Brazil, living exclusively inside bromeliads on a high elevation rocky outcrop and with an extent of occurrence of less than ca. 0.5 km^2^. *Crossodactylodes itambe* is included in the Brazilian Conservation Action Plan for amphibians at the Espinhaço Mountain Range^37^, which recommends the implementation of long-term monitoring studies for threatened species that are rare and elusive. Our aim was to design a monitoring protocol that improves the chance of detecting a population change, which could also allow better allocation of survey effort and financial resources. We therefore addressed three questions fundamental to any monitoring programme: (1) Is the currently used sampling design providing precise estimates of occupancy and detectability? (2) Is this sampling design providing sufficient power to detect changes in occupancy over time? (3) How can we improve statistical power to detect small changes in populations? The bromeliad-frog system therefore provides an opportunity to explore issues of sampling and statistical power that would prove unwieldy on a larger landscape scale and we present a rigorous assessment that could benefit future monitoring programmes in their earlier stages.

## Methods

### Study system and sampling design

The Itambe summit is the highest point of the Espinhaço Mountain Range at 2062 m above sea level (a.s.l.) and is located in South-eastern Brazil, in Minas Gerais state. The area is characterized by open field habitats with vegetation growing in humid rocky outcrops. *Crossodactylodes itambe* is restricted to 1800 m a.s.l. and occupies a single species of bromeliad (*Vriesea medusa*), where it spend its entire life cycle^35^. Individuals have never been observed outside bromeliads and are mostly inactive inside the plant (Barata I. M., *manuscript in preparation*). Although territorial behaviour may occur^36^, dispersal may be confined to rain storms when it is difficult to make observations. Considering field observations, life history of the genus and the small size of individuals^35,36^ we believe that species dispersal capability is low and we therefore considered individual bromeliads as independent sampling sites. To ensure independence within and between survey periods, sampled bromeliads were at least 25 m apart. We divided the study area into three altitudinal zones: low (1704–1815 m a.s.l.), medium (1838–1925 m a.s.l.) and high (1998–2060 m a.s.l.) – delimited by the topography of the area and the species’ distribution. Within these zones, we randomly tagged individual bromeliads using numbered labels that allowed repeated visits. In 2014 we tagged 123 bromeliads, and we added 20 new bromeliads in the following year. In 2015, the 143 sampling sites were equally distributed among the altitudinal zones (47 bromeliads at high elevation, 48 at the medium and low zones).

In February 2014 we surveyed our sites on four sampling occasions (four consecutive nights). We considered this year as a pilot study to test the feasibility of our sampling design. The following year, we increased the number of sampling occasions (4–6 consecutive nights) and repeated this survey effort monthly from February to May, representing wet and dry seasons. Monthly surveys were separated by 15–25 days. We surveyed frogs using visual encounter surveys, developed by two teams of two observers each, starting after dusk. To standardize our surveys, only one person of each team was allowed to record species presence/absence, and both received training in observing the target species. We recorded species presence and absence, using adults, juveniles and tadpoles as evidence of species presence at a site.

We considered repeated nights as independent sampling occasions. For each month, we assumed individuals did not leave the site between sampling occasions and we targeted bromeliads with the numbered labels. However, some tagged plants had neighbouring bromeliads touching their edges (forming a patch of several conspicuous bromeliads) and we considered that frogs might have moved to a neighbouring bromeliad between the monthly surveys. Therefore, we also searched for frogs inside the neighbouring bromeliads. On every sampling occasion, we first surveyed the tagged site recording species presence/absence, and we then searched neighbouring bromeliads, irrespective of presence/absence in the tagged site.

### Modelling species occupancy

Occupancy modelling is based on the patterns of detection and non-detection and estimates both site occupancy (i.e., the probability of a randomly selected site being occupied by a species) and detection probabilities, accounting for imperfect detection^14^. Some assumptions are required for the standard single season occupancy model^13,14^. The model assumes that there are no false detections, but failure to detect the species indicates either that the site is truly unoccupied or that it is occupied but the species was missed during the survey. Also, the detection of a species at a site is independent of detections of the species at all other sites. Finally, each site is either occupied through the entire season or unoccupied throughout. To avoid violating these assumptions we used detection histories from tagged and/or neighbouring bromeliads, and analysed data for individual months, wet and dry seasons and complete years.

For monthly datasets (one month in 2014; four months in 2015), we estimated occupancy and detection probabilities using the detection histories from tagged sites only (assuming individuals did not leave the site during sampling occasions). However, because of the time interval between months, we assumed that individuals might have moved within the patch from one survey to the other. In this last scenario, for the 2015 dataset, we grouped detection histories from tagged and neighbouring bromeliads into a single dataset: (1) to estimate parameters for seasons, where we grouped two months of data (wet season: Feb-Mar; dry season: Apr–May); and (2) to compare estimated occupancy and detectability between years, grouping all four monthly surveys (Feb–May 2015). Therefore, to compare variation in occupancy and detectability between months, seasons and years, we fitted single season models which assumed a constant occupancy and detection probability across sites (hereafter, constant models). We also estimated parameters for each altitudinal zone to account for changes related to elevation. Because we aimed to explore aspects of sampling design (and also to simplify the analysis), we did not use the dynamic occupancy model, which could estimate colonization and extinction processes in the population^13^.

The occupancy model can accommodate covariates which may be either site or survey specific^13,14^. We also developed models testing a priori hypotheses focusing on the drivers of occupancy and detectability (based on species traits and expert knowledge). Because the February 2014 dataset was considered a pilot study, we only tested models for the complete 2015 dataset. We incorporated covariates potentially related to sampling design. We used survey-specific covariates: time of observation (given by time after dusk); observer experience (low or high, given as a categorical variable), and site-specific covariates: number of leaves in bromeliad; number of neighbours; size of bromeliad (given by height x width); volume of rosette (given by height x width); and elevation (meters a.s.l.). Correlated covariates were excluded from the model selection.

We used a stepwise model selection approach to build our model, where we combined covariates for both detectability and occupancy. We first established models that included only covariates of detectability (in this case, occupancy was kept constant). We then selected the best models and incorporated covariates of occupancy, combining them with detectability covariates previously indicated by model selection. We used the Akaike Information Criterion (AIC) to rank candidate models and to calculate Akaike weights^38^. Models were ranked by their AIC (the model with the lowest AIC value having best fit) and weighted as the probability of being the best model in the set, indicating relative support of a model. We selected the best models based on ΔAIC: models with ΔAIC < 2 had strong support while models with a ΔAIC of >2 were considered to have less support^38^. Occupancy models and model selection were performed in R^39^, using the package Unmarked^40^.

### Optimal survey design and statistical power

We used estimates of occupancy and detectability from constant models to explore the number of surveys required to detect the species at a given site and to compare the statistical power of sampling designs from our pilot study and the following year. However, because models with covariates were fitted for the 2015 dataset, we used occupancy and detectability estimates from our best model to calculate improvements in power in relation to sampling design.

We first evaluated the survey design used in our pilot study. We used estimated detection probabilities to predict the minimum number of occasions (K) required to determine that the species is truly absent from a site, using the expression^19–21^:

$${\rm{K}}=\,\mathrm{log}(1-{p}^{\ast })/\,\mathrm{log}(1-p),$$

where *p* denotes the detection probability, and p* is the desired probability of detecting the species at an occupied site on at least one of the K visits (set to be 0.8, 0.9 and 0.95). We applied the predicted number of visits in our surveys in the following year, and performed the same calculations for the 2015 monthly dataset to account for changes in the value of K required from one survey to the other.

We then used the predicted K to investigate the precision of our estimates as a criterion for sampling design. We used R functions available in Guillera-Arroita *et al*.^17^ that simulate data for a given set of parameter values and sampling designs to allow the quality of the estimators to be assessed under different combinations of survey effort. These functions can be applied to the single season single-species occupancy models with constant probabilities, and firstly, generate simulated histories, calculating the corresponding maximum-likelihood parameter estimates (MLE) of occupancy and detectability and evaluating the estimator performance. Secondly, the functions display the distribution of the MLEs obtained for the given design and values of occupancy and detectability^17^. To evaluate the performance of our initial sampling design, using occupancy and detectability from our pilot study, we simulated single season occupancy models varying the number of sampling occasions (according to the previous calculations of K) and with a fixed number of sites from our pilot study. We also performed simulations with different levels of survey effort (i.e., a combination of varying number of occasions and sites) to explore an ‘optimum survey design’, which achieves good precision of estimated parameters with only a few visits.

We proceeded to evaluate the statistical power of our sampling design. We first compared statistical power between years (2014 and 2015 datasets). Using occupancy and detectability estimates, we calculated power as a function of the change in occupancy. Power is related to error types, the effect size, the sample size and the sample variance^30^. In this study we considered the change in occupancy as the effect size, which compares the state of occupancy at two different points in time and represents an increase or decrease from the initial state (for example, an effect size of 30% means that occupancy decreased from 70% to 40%). Statistical tests can give rise to two types of error: a Type I error occurs if a change is detected when in reality there is no change, and a Type II error arises when the test fails to detect a change that is present. The probability of a Type I error is denoted by *α* and the probability of a Type II error by *β*. A significance level conventionally chosen is 0.05 for *α* and 0.8 for *β* (known as the five-eighty convention^41^). However, since power is given by G = 1 − *β*, levels of significance should reflect the relative seriousness of committing Type I and II errors^18,31^. Considering our monitoring goal, we assumed that making a Type II error would be highly costly (i.e., not detecting a change in occupancy when there is one) and we therefore used higher levels of *α* (0.1).

For power analysis we used calculations with a Wald test on the probabilistic scale^18^. We used estimated parameters from our best model. We explored power as a function of survey effort and observer experience, varying the number of occasions, sites and detectability under different effect sizes (from 0.15 to 0.3). To complete our analysis, we investigated statistical power varying the number of sites (50–300 sites), including our current sampling design (143 sites). For this analysis, we built a two-tailed power curve as a function of effect size, keeping occupancy, detectability and the number of occasions constant. Finally, to demonstrate the influence of significance levels in sampling design, we calculated the number of sites needed to achieve a given power (from 0.8 to 0.95) under different levels of significance (from 0.05 to 0.2). We also performed a Wald test (5000 iterations) to verify the actual performance under different numbers of sites. We used this approach to explore the power of sampling designs and evaluate the pros and cons of different survey efforts when suggesting a long-term monitoring protocol.

## Results

**Figure 1:**
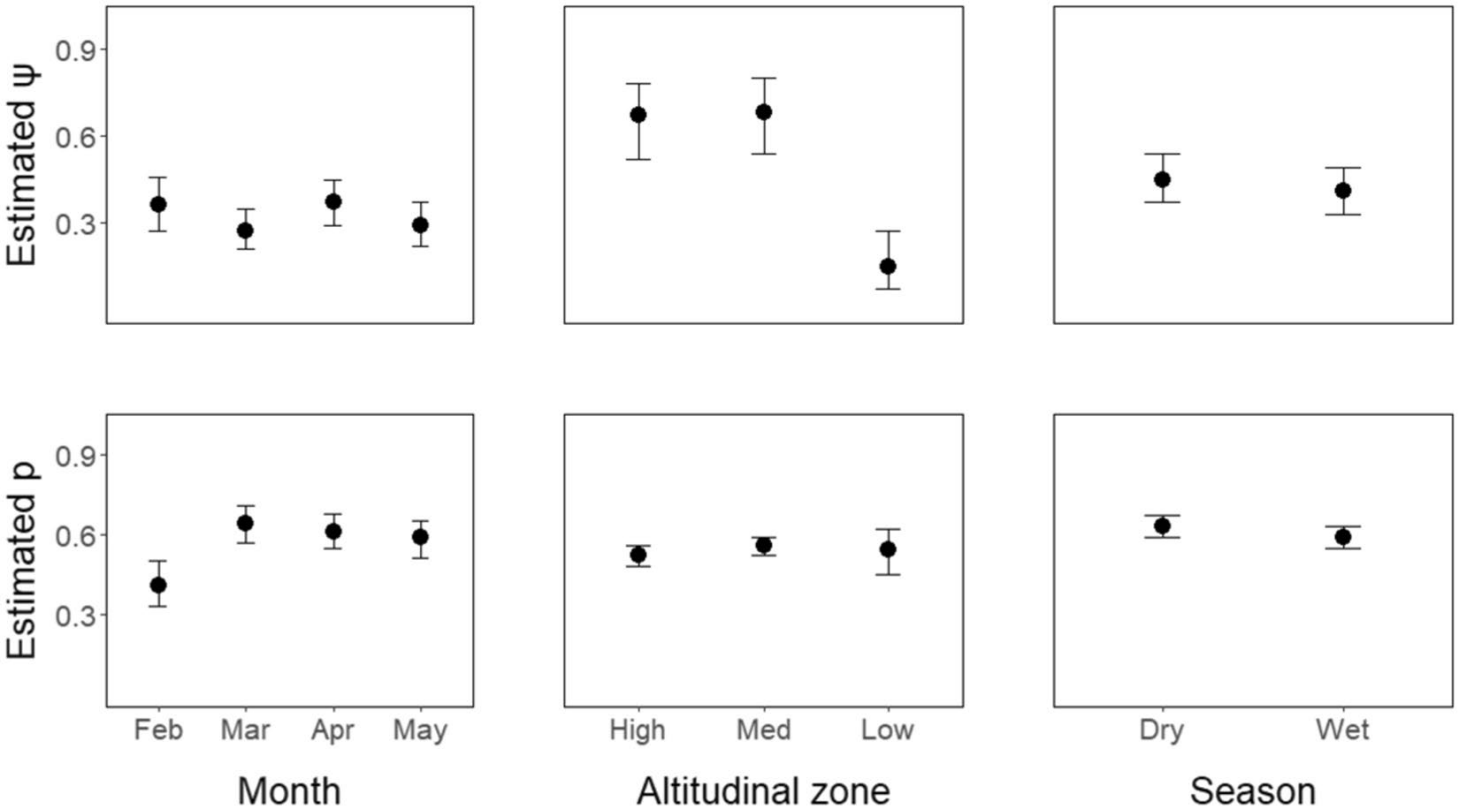
Estimated occupancy (ψ) and detection (*p*) probabilities for 2015 monthly dataset using site only, varying with months (February to May), altitudinal zone (high, medium and low) and season (dry and wet). Vertical lines are 95% confidence intervals.

*Crossodactylodes itambe* had a relatively high detection probability, with little variation in relation to elevation, among months and between seasons (Fig. 1; Supplementary Table S1) – although Fig. 1 does suggest lower detectability in February 2015. Overall, there was a 40-65% chance of detecting the species in a bromeliad if that site was occupied. Occupancy also did not vary between months or seasons (Fig. 1; Supplementary Table S1), but varied with elevation. While 66% of bromeliads were estimated to be occupied at high and medium elevation, only 14% were estimated to be occupied at the lowest altitude. Stepwise model selection indicated that observer experience was the best covariate explaining detectability (AIC weight 0.96). We tested six models combining observer experience with occupancy covariates (size of bromeliad and elevation), but also including a constant model (Supplementary Table S2). The best-fitting model had elevation as an important covariate explaining occupancy (ΔAIC < 2, AIC weight 1). Detection probability was explained by observer experience, and detectability differed significantly between observers (observer A, experienced: 0.61, 95% CI 0.57–0.65; observer B, inexperienced: 0.38, 95% CI 0.32–0.43). Other models had little support and were unlikely to explain estimated parameters (Supplementary Table S2).

**Figure 2:**
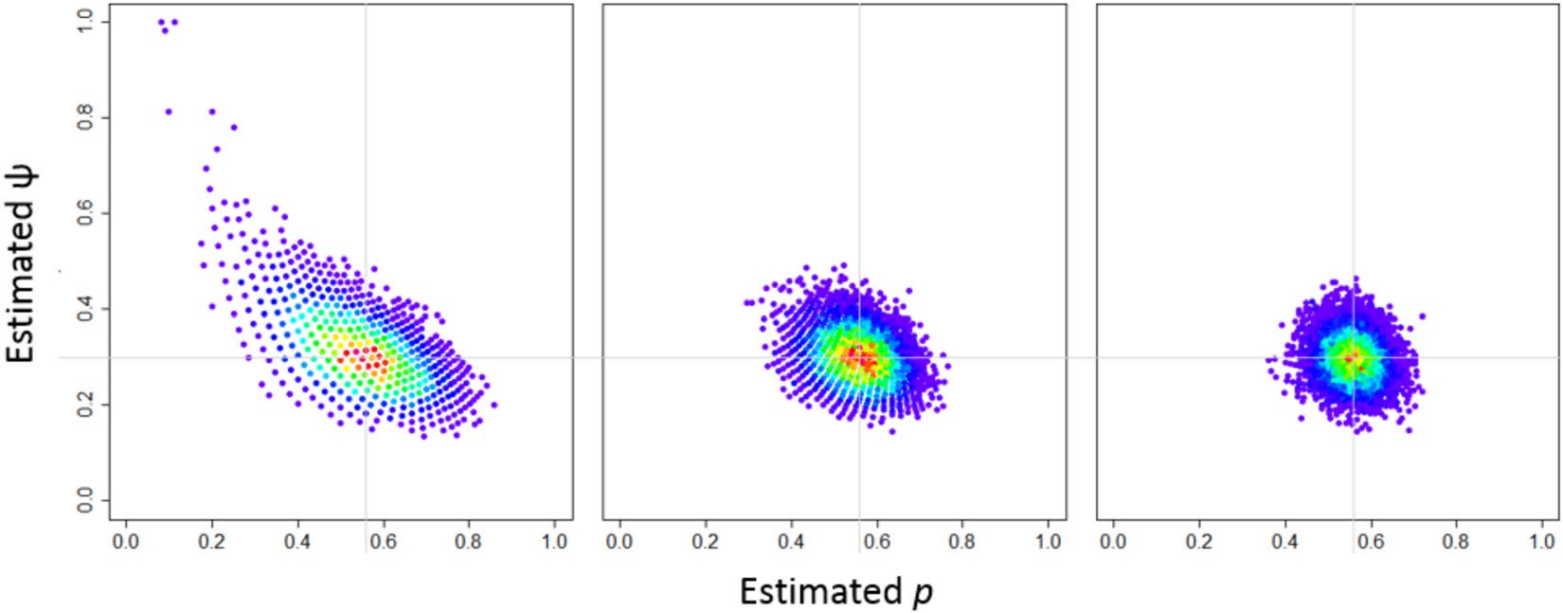
Distribution of the maximum likelihood estimates (MLE) for 2014 dataset (ψ = 0.3, *p* = 0.56, 10000 iterations) with varying number of visits according to predicted number of occasions (2, 3 and 4 visits; previous calculations) and with a constant number of sites (S = 123).

The number of sampling occasions required to determine species presence at occupied sites varied according to the desired confidence level (Supplementary Table S3). Based on our estimates of occupancy and detectability, the required number of visits for our 2014 pilot study varied from two to four. Simulations showed that three visits are enough to provide reliable estimates of occupancy and detectability (Fig. 2). The required number of visits in the 2015 dataset varied from two to six (Supplementary Table S3). Further simulations demonstrated that a reduced number of sites (n = 50) would require a large number of sampling occasions to improve precision (Supplementary Fig. S1). On the other hand, a large sample (with 150 sites) would require as few as two sampling occasions to produce reliable estimates. In any scenario, there is a slight improvement in precision after four sampling occasions (Supplementary Fig. S1).

**Figure 3:**
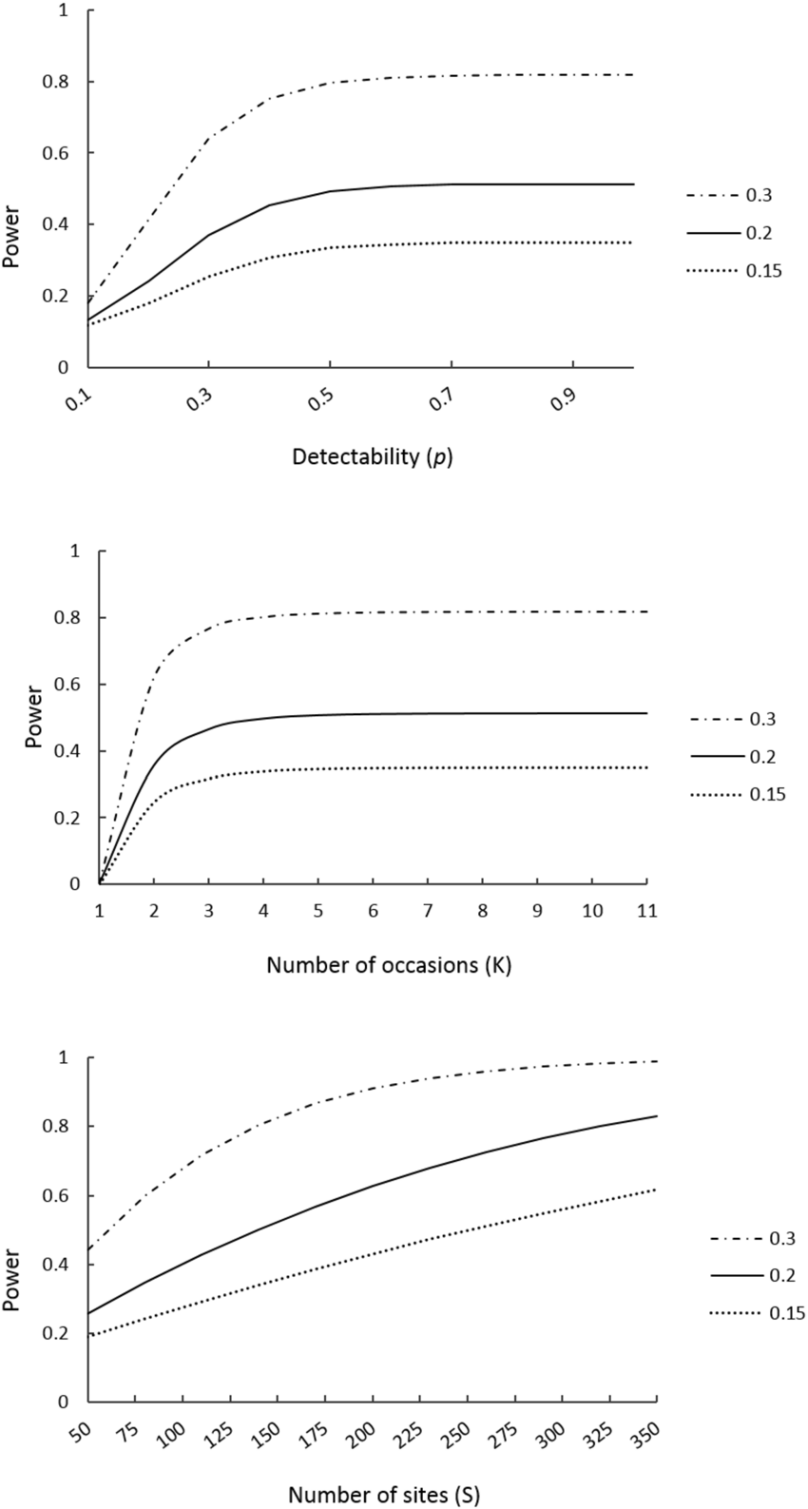
Statistical power for different changes in occupancy (effect sizes of 0.3, 0.2 and 0.15) with respect to species detectability (*p*), number of sampling occasions (K) and number of sampling sites (S).

**Figure 4:**
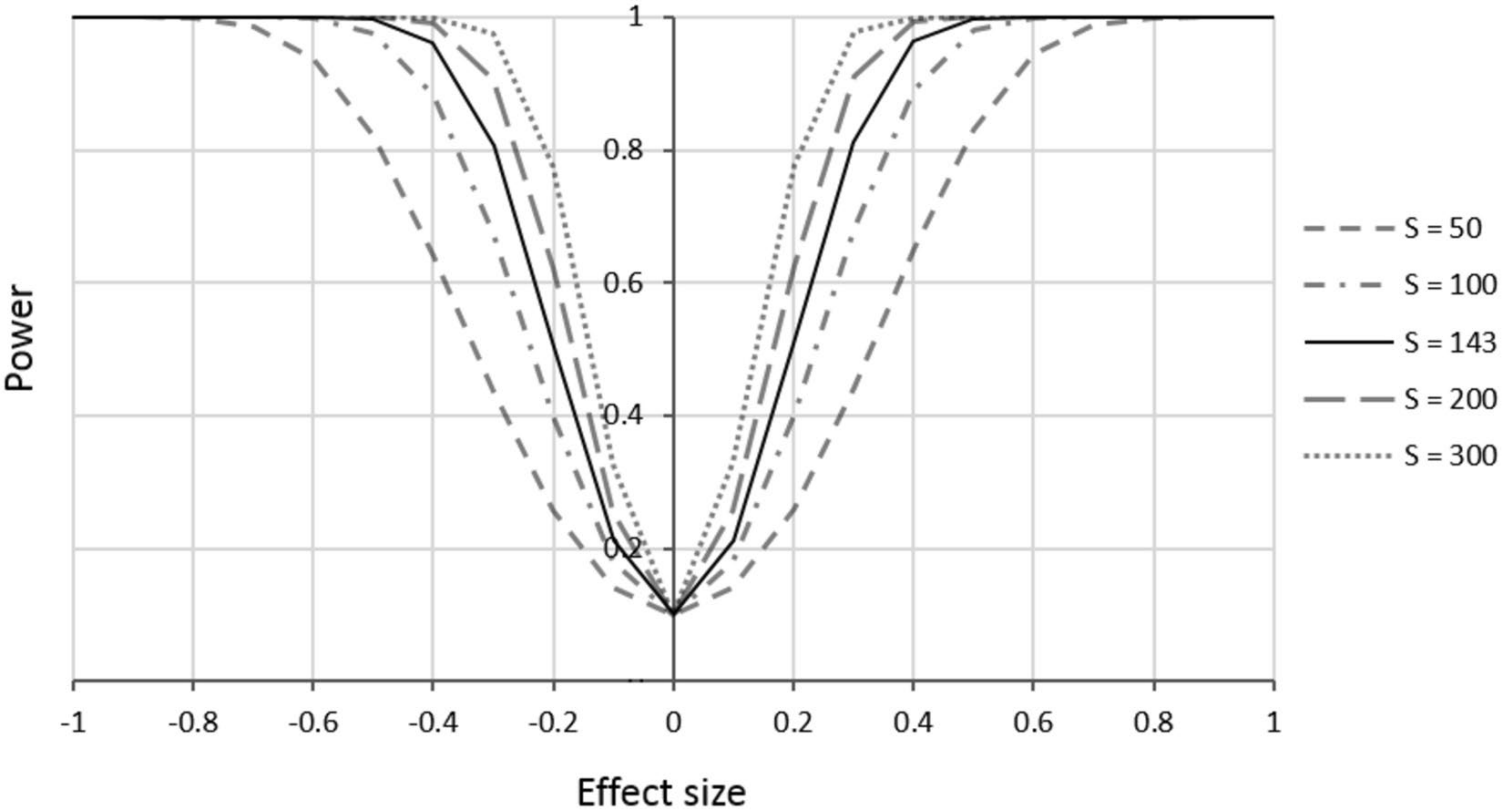
Statistical power as function of change in occupancy (effect size) under different sampling designs, based on estimates of best model psi(alt)p(obs) for 2015 dataset (ψ = 0.49; *p* = 0.61; α = 0.1). S = number of sites surveyed.

There was an increase in statistical power between years (Supplementary Fig. S2). While our pilot study (with 123 sites and 4 visits) had an 80% chance of detecting a 50% change in occupancy, in 2015 our increased survey effort had the same chance of detecting a 30% change in occupancy. Calculations varying the number of sampling sites, sampling occasions and detectability showed how the power to detect a smaller change in occupancy (from 15–30%) could be increased. There was constant statistical power after three visits and the power to detect a change did not increase with detectability over 0.5 (Fig. 3). Nonetheless, we observed an increase in power by increasing the number of sites (Fig. 3). The sampling design currently used had 82% power to detect a change of 30% in occupancy; smaller changes had less statistical power, with 53% and 36% chances to detect changes of 20% and 15%, respectively. We found that doubling the number of sites would detect a 20% change in occupancy, with the same statistical power (0.8; Fig. 4). However, the number of sites needed depended on the significance level and the effect size (Supplementary Table S4). For example, to detect a 15% change in occupancy in *C. itambe* at a significance level of 0.1 would require 565 sampling sites.

## Discussion

Although cost-effective sampling designs can be based on simulated data^15,17^, many aspects require customization using real data and sampling needs^10^. Sampling designs depend on detection probabilities^15,42^; in the case of amphibians, detection may change considerably in relation to season, such as an increase of frogs during rainy periods^43^ or a decrease of salamanders over the summer^26^. We observed little variation in detectability between seasons or among months (except for February, when detectability was slightly lower), which means that *C. itambe* can be detected regardless of the sampling period. Potential seasonal variation should be accounted for when designing surveys, especially for species that are detectable only during short time-frames (e.g., seasonal breeding frogs). Surveys should therefore target periods when detectability is likely to be high^20,26^, particularly when dealing with elusive species.

The detection probability of *C. itambe* was strongly influenced by observer expertise, which therefore had an effect on our sampling design. Less experienced observers need to carry out a larger number of survey visits to compensate for their lower species detection rates. In our case, species misidentifications by different observers – which can lead to false positives and introduce bias in occupancy estimates^44–46^ – were considered unlikely. Other amphibian species using bromeliads in the study area are rare; indeed, other frog species are morphologically distinct from *C. itambe* and do not use the bromeliads for breeding. Additionally, because detectability is influenced by abundance^47,48^, local density of individuals is also relevant to observer experience. Volunteers may fail to detect low-density populations of invasive pests when compared to experts^45^, which might have implications for designing surveys for rare and cryptic species. Despite being considered range restricted, *C. itambe* showed moderate levels of occupancy – meaning the species is rare, but with high local occurrence – providing a good opportunity for reliable monitoring at the local scale.

Observer experience is an important source of sampling variation^45,49^ and accounting for differences in detectability among observers can improve survey design and avoid inefficient sampling^50–52^. Although differences between observers have been previously reported^47–51^, the impact of such variation on the quality of biodiversity data is poorly understood^53^. We attempted to minimize data heterogeneity by training the observers, who could also gain experience with time. Although training can reduce bias and variability^49^, in our case, even after training, there remained a difference in detection when accounting for expertise. Therefore, training did not eliminate the importance of experience in monitoring the species. Consequently, inter-observer variation should be acknowledged when designing a survey and included in the model selection when estimating the parameters of interest.

When designing surveys, the trade-off between the number of sampling occasions and the number of sites needs to be assessed. While a reduced number of sites required a higher number of sampling occasions to maintain precision and accuracy of the parameters we estimated, an increased number of sites needed only a few visits. Precision is gained by increasing sampling occasions^15,23,42,54^ and, as observed in other amphibians, the number of visits required increased with the level of certainty needed^19,20,28^. Thus the minimum number of sampling occasions must (1) ensure recorded absences from a site are reliable; and (2) deliver precise estimates of occupancy and detectability. However, there was a limit to increasing precision with little improvement after three to four visits, presumably because there is no real uncertainty remaining about whether the site is occupied. In our case, relatively few visits were sufficient to estimate parameters with good precision, which can reduce the costs of the monitoring programme.

In some respects, *C*. *itambe* may be an unusual model for a rare and threatened species. Estimates of amphibian detection are frequently low^20,22,43,55^ and this is particularly challenging for population monitoring. Although strong inferences on population trends are mostly needed for rare and cryptic species, these are the very taxa that display low detectability or occupancy rates (or both). Amphibian detectability can be improved by conducting surveys with multiple observers or repeating sampling occasions in a single night^13^, increasing the number of traps and/or reducing the sampling area^43^, combining different sampling methods^20,22,56^ or surveying under ideal weather conditions^19^. However, it may be more costly to obtain precise estimates for rare and cryptic species because of the increased sampling effort needed. Therefore, explorations of sampling design should be developed during the early stages of a monitoring programme and designed to be both species and habitat specific.

If a monitoring programme aims to detect changes in a given population, the sampling design should be able to distinguish real trends from stochastic fluctuations^5^. As in other studies, power increased with sample size, but also depended on the level of significance and the effect size considered^9,57^. Appropriate levels for *α* and *β* depend on the goals of a study and should not be set arbitrarily^41^. Our results show the impact of this choice on the sampling design. For monitoring programmes, we suggest that statistical power should be investigated with *α* = 0.1 as previously applied in sampling designs^27^ to avoid the negative consequences of not detecting a change in occupancy when in fact there is one (i.e., committing a Type II error). Although relatively few visits were required to deliver good precision in our study system, the same sampling design can yield good statistical power, but it was limited to detecting changes of at least 30% in occupancy. In our case, the sampling effort needed to detect a 15% change would require an unrealistic number of sampling sites. Nonetheless, we must consider the effect size expected to be seen when monitoring a target species – which should be based on previous knowledge, such as pre-existing data or ecological theory^31^. Although the effect size that can be detected by our current sampling design is not ideal, we considered it acceptable for this population.

Very often sampling designs are unlikely to provide sufficient power to detect small changes in estimated parameters^9,27,28^ especially for less detectable species^58^. Statistical power to detect small changes can be increased by changing the sampling design, usually (if not always) by increasing sampling effort^5,9,22,25,27–29,59^. In our case, increasing the number of sampling occasions had only a small effect on statistical power. In fact, relatively few observations are needed to maximize the power to detect trends^5^ and there is no improvement after a given number of occasions^28,29,59^. For amphibians, detectability has been previously shown to affect the power to detect occupancy changes^22^. However, after detectability reached 0.5 we found no further increase in statistical power. In the case of *C. itambe*, increasing the number of sampling sites is the only strategy to improve power to detect small changes, which was also suggested for bats^8^, amphibians^22,25^, reptiles^28^ and large mammals^60^.

As demonstrated by the improved statistical power between years, higher initial occupancy probability yields larger statistical power^18^ – an effect previously shown for other amphibians^22,28^. Species with lower initial occupancy rates, such as rare species, will therefore require more sites^22^. The definition of sampling sites can vary from a single unit to a patch of potential breeding habitat^10^. Because spatial correlation can reduce power^60^, distance between sites must respect species distribution, home range and dispersal capabilities. If sites are close, surveys can be done by multiple observers on the same night – a design that could be applied for pond-breeding amphibians with moderate detectability. For territorial frogs, sites could be closely located (e.g. sub-transects in the same stream) and visited during a short survey window, when detectability is higher. The number of sites can also be increased by placing automated recording units and/or increasing the number of traps. A removal sampling design can be applied (although this might be less robust to model assumptions^15^), as well as a double sampling design, for which a high detection probability is required^15^. Nevertheless, as we demonstrated here, for species restricted to a small number of remaining sites, obtaining sufficient statistical power to distinguish real population changes from natural fluctuations may be an unachievable goal.

In many cases, increasing the number of sites may pose a problem for monitoring species with highly restricted distributions and which occur at relatively few sites. Even when the sampling design provides precise estimates and reliable power, we can only realistically detect moderate to large declines in the population and smaller changes will not be detected. Unless there is high abundance and moderate initial occupancy, rare and cryptic species will be particularly challenging when it comes to detecting population changes. Our data show the importance of considering inter-observer variation in detection probabilities and we emphasize that future monitoring should consider the role of observer variability when estimating occupancy and detectability. For monitoring programmes in their initial stages, we recommend a pilot study to optimize the sampling design of the main study. Although we have used specific data from a single case study, the same modelling and calculations can be applied to any target species. This can be particularly useful for targeted species in the Brazilian Conservation Action Plan for threatened amphibians at the Espinhaço Mountain Range^37^, where long-term monitoring studies are proposed. Some existing tools are available to evaluate the bias and variance of the estimated parameters from a given sampling design (e.g., GenPres^16^ and SODA^17^), which should make the analytical process straightforward for conservation practitioners. Failing to deliver precise estimates and appropriate levels of statistical power will lead to cost-ineffective surveys designs as well as spurious conclusions about population trends.

### Data availability

The dataset analysed during the current study are available from the corresponding author on reasonable request.

## Electronic supplementary material

Supplementary information
