# Supplementary material for: The power of monitoring: optimizing survey designs to detect occupancy changes in a rare amphibian population
Source: Sci Rep. 2017 Nov 28;7:16491. doi: 10.1038/s41598-017-16534-8 (PMC5705711; doi:10.1038/s41598-017-16534-8)
Supplement: Supplementary file 1 — Supplementary information [file 41598_2017_16534_MOESM1_ESM.pdf]

The power of monitoring: optimizing survey designs to detect occupancy changes in a rare amphibian population

Izabela M. Barata[1*], Richard A. Griffiths[1] and Martin S. Ridout[2]

[1] *Durrell Institute of Conservation and Ecology, School of Anthropology and Conservation, University of Kent, Canterbury, Kent, CT2 7NR, UK*

[2] *National Centre for Statistical Ecology, School of Mathematics, Statistics and Actuarial Science, University of Kent, Canterbury, Kent, CT2 7NF, UK*

* Corresponding author: ib208@kent.ac.uk

Supplementary information

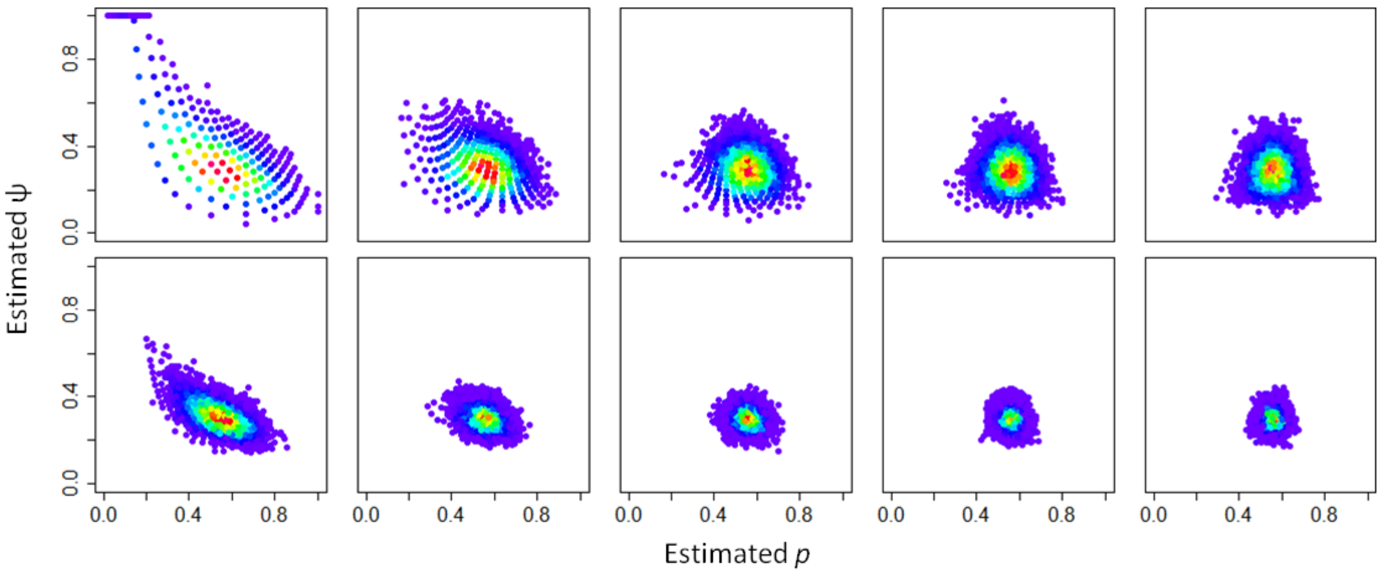

Figure S1. Distribution of the maximum likelihood estimates with varying number of occasions (2–6 visits) and with a constant number of sites (first line, S = 50; second line S = 150). Estimates are based on constant model psi(.)p(.) using detection history of sites and neighbours from 2014 dataset, where $\psi = 0.3$, $p = 0.56$ (10000 iterations).

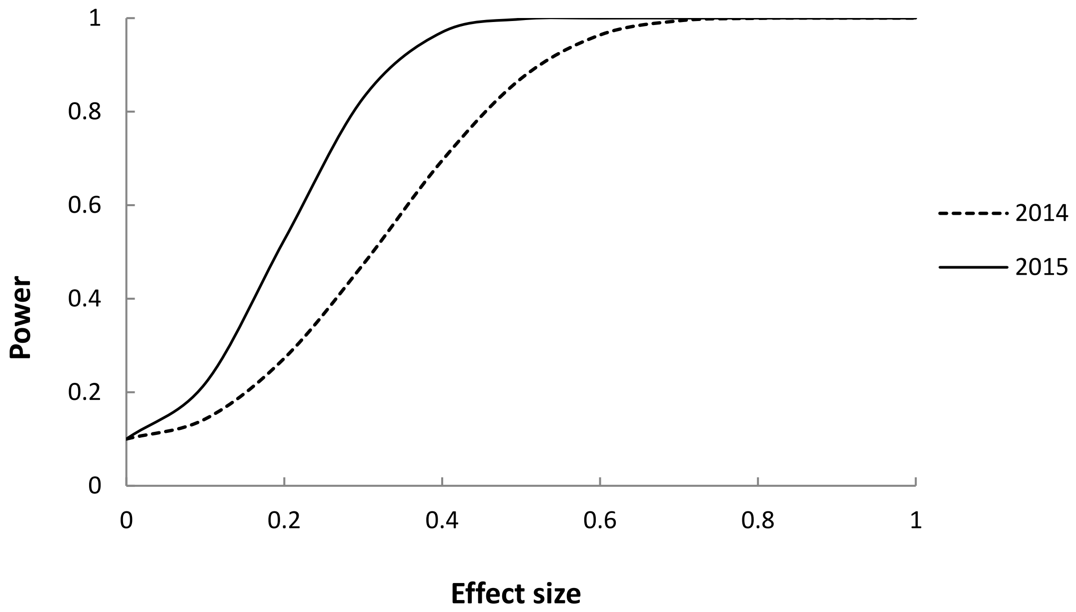

Figure S2. Statistical power between years in relation to effect size (absolute change in occupancy) for 2014 (123 sampling sites, 4 sampling occasions, $\psi = 0.3$, $p = 0.56$) and 2015 (143 sampling sites, 21 sampling occasions, $\psi = 0.49$, $p = 0.54$) ($\alpha = 0.1$ for both datasets).

Table S1. Naïve occupancy, estimated occupancy (ψ) and detection probability (p) based on a constant model psi(.)p(.) for **A)** 2014 and 2015 monthly dataset using site only and **B)** for 2015 complete dataset using patch data (sites and neighbours), giving 95% confidence interval (CI), number of sampling sites (S) and number of occasions (K); where $p*$ is the probability of detecting a species at an occupied site at least once, given by: $p^* = 1 - (1-p)^K$

A)

	naïve ψ	ψ	CI	p	CI	$p*$	S	K
**2014 monthly dataset**								
FEB	0.27	0.30	0.22 – 0.4	0.56	0.45 – 0.66	0.96	123	4
**2015 monthly dataset**								
FEB	0.25	0.36	0.27 – 0.46	0.41	0.33 – 0.50	0.93	131	5
MAR	0.25	0.27	0.21 – 0.35	0.64	0.57 – 0.71	1.00	143	6
APR	0.36	0.37	0.29 – 0.45	0.62	0.55 – 0.68	0.99	143	5
MAY	0.29	0.29	0.22 – 0.37	0.59	0.51 – 0.65	0.99	143	5

B)

	Naïve ψ	ψ	CI	p	CI	$p*$	S	K
**2015 complete dataset**								
2015	0.50	0.50	0.41 – 0.58	0.54	0.51 – 0.57	1.00	143	21
**Season**								
DRY	0.45	0.45	0.37 – 0.54	0.63	0.59 – 0.67	1.00	143	11
WET	0.40	0.41	0.33 – 0.59	0.59	0.55 – 0.63	1.00	143	10
**Gradient**								
HIGH	0.67	0.67	0.52 – 0.78	0.52	0.48 – 0.56	1.00	48	19
MED	0.68	0.68	0.54 – 0.80	0.56	0.52 – 0.59	1.00	47	20
LOW	0.14	0.14	0.07 – 0.27	0.54	0.45 – 0.62	1.00	48	18

Table S2. Fitted models for 2015 complete dataset (detection history from sites and neighbouring bromeliads): A) with covariates for detectability (*p*) and constant occupancy (psi); and B) including covariates for both parameters. N Pars = number of parameters; ΔAIC is the difference between the model with the lowest AIC and the given model; AIC *w* is AIC weight. Constant model is also included, given by psi(.)p(.); covariates of detectability are: obs = observer experience, neig = number of neighbours in the patch, leaf = number of leaves in bromeliad, time = time of observation, vol = volume of rosette; covariates of occupancy are: alt = elevation, size = size of bromeliad.

A)

Model	N Pars	AIC	ΔAIC	AIC *w*	Cumulative Weight
psi(.)p(obs)	3	1906.82	0.00	0.96	0.96
psi(.)p(neig)	3	1912.98	6.16	0.044	1.00
psi(.)p(.)	2	1924.19	17.37	<0.001	1.00
psi(.)p(leaf)	3	1924.23	17.42	<0.001	1.00
psi(.)p(time)	3	1925.29	18.48	<0.001	1.00
psi(.)p(vol)	3	1925.58	18.76	<0.001	1.00

B)

Model	N Pars	AIC	ΔAIC	AIC *w*	Cumulative Weight
psi(alt)p(obs)	4	1882.82	0.00	1	1.00
psi(alt)p(.)	3	1900.19	17.37	<0.001	1.00
psi(size)p(obs)	4	1904.36	21.54	<0.001	1.00
psi(.)p(obs)	3	1906.82	24.00	<0.001	1.00
psi(size)p(.)	3	1921.74	38.92	<0.001	1.00
psi(.)p(.)	2	1924.19	41.37	<0.001	1.00

Table S3. Number of surveys required to determine species presence at occupied site given 80%, 90% or 95% certainty. S = number of sites surveyed; K =number of visits; ψ = estimated occupancy; *p* = estimated detectability for each dataset. Estimates are based on constant model psi(.)p(.) using monthly detection history of sites only. Minimum and maximum number of surveys needed are given in bold.

	S	K	ψ	*p*	N survey needed		
					0.8	0.9	0.95
2014	123	4	0.3	0.56	2.0	2.8	3.6
FEB	131	5	0.36	0.41	3.1	4.4	**5.7**
MAR	143	6	0.27	0.64	**1.6**	2.3	2.9
APR	143	5	0.37	0.62	1.7	2.4	3.1
MAY	143	5	0.37	0.62	1.7	2.4	3.1

Table S4. Number of sampling sites needed to achieve a given power (G; from 80–95%), considering different effect sizes (R; from 0.5–0.15) and varying significance level (α; from 0.05–0.2) (based on estimates of best model for 2015 complete dataset: $\psi = 0.49$, $p = 0.61$, 21 occasions, 143 sites).

G	α	R			
		0.5	0.3	0.2	0.15
0.8	0.05	57	173	400	717
	0.1	45	136	315	565
	0.2	33	100	230	412
0.85	0.05	66	198	457	820
	0.1	53	159	366	656
	0.2	39	119	274	491
0.9	0.05	77	232	535	959
	0.1	63	189	436	782
	0.2	48	145	334	600
0.95	0.05	95	286	661	1186
	0.1	79	239	551	988
	0.2	63	189	436	782
